# Evidence of the Link between Stroma Remodeling and Prostate Cancer Prognosis

**DOI:** 10.3390/cancers16183215

**Published:** 2024-09-21

**Authors:** Davide Vecchiotti, Letizia Clementi, Emanuele Cornacchia, Mauro Di Vito Nolfi, Daniela Verzella, Daria Capece, Francesca Zazzeroni, Adriano Angelucci

**Affiliations:** Department of Biotechnological and Applied Clinical Sciences, University of L’Aquila, 67100 L’Aquila, Italy

**Keywords:** reactive stroma, cancer-associated fibroblasts, myofibroblasts, inflammation, extracellular matrix, energy metabolism

## Abstract

**Simple Summary:**

Prostate cancer (PCa) is among the most common cancers. While PCa is frequently diagnosed in elderly men as a slow-growing, low-risk disease, in about 10–15% of cases, it can become a life-threatening danger. Unfortunately, biomarkers able to discriminate indolent prostatic tumors from aggressive forms are lacking, and watchful waiting remains one of the best options. However, available data support the hypothesis that distinctive biological characteristics of PCa stroma can contribute to cancer progression in a clinically relevant way. According to the current view, tissue alterations induced by tumor growth affect both metabolism of resident normal cells and composition of the extracellular matrix and are able also to recruit cells from circulation. Here, we seek to respond to the challenge of identifying stroma-associated biomarkers that may be relevant to assist prognostic decisions in PCa patients.

**Abstract:**

Prostate cancer (PCa), the most commonly diagnosed cancer in men worldwide, is particularly challenging for oncologists when a precise prognosis needs to be established. Indeed, the entire clinical management in PCa has important drawbacks, generating an intense debate concerning the possibility to individuate molecular biomarkers able to avoid overtreatment in patients with pathological indolent cancers. To date, the paradigmatic change in the view of cancer pathogenesis prompts to look for prognostic biomarkers not only in cancer epithelial cells but also in the tumor microenvironment. PCa ecology has been defined with increasing details in the last few years, and a number of promising key markers associated with the reactive stroma are now available. Here, we provide an updated description of the most biologically significant and cited prognosis-oriented microenvironment biomarkers derived from the main reactive processes during PCa pathogenesis: tissue adaptations, inflammatory response and metabolic reprogramming. Proposed biomarkers include factors involved in stromal cell differentiation, cancer-normal cell crosstalk, angiogenesis, extracellular matrix remodeling and energy metabolism.

## 1. Searching New Biomarkers in Prostate Cancer Stroma

Intense efforts have been performed by scientists in the field of prostate cancer (PCa) to identify new biomarkers able to discriminate PCa from benign prostatic conditions (diagnostic biomarker), between indolent and aggressive tumors (prognostic biomarker) and between responsive and unresponsive patients (predictive biomarker). The current diagnostic methods for PCa detection, including digital rectal exam, serum prostate-specific antigen (PSA) and derivatives (PSA density, PSA velocity, % between total and free PSAs) and transrectal ultrasound image-guided biopsy, lack the necessary specificity/sensitivity or are limited by inter-observer variability [1]. Indeed, it is estimated that the current diagnostic methods may miss up to 30% of clinically significant PCa [2]. In addition, the identification of new biomarkers that support physicians in their decision-making activity could help in identifying those patients who are suitable for active surveillance, avoiding unnecessary or invasive treatment (e.g., radical prostatectomy (RP), hormone therapy, chemotherapy) and ensuring a more precise clinical management [3,4].

Initially, carcinogenesis and cancer progression were considered a consequence of pathological alterations affecting only cancer cells. This consideration was abandoned when it was ascertained that the tumor microenvironment (TME) provides indispensable fertile soil for cancer growth. A more recent and radical suggestion envisions that the TME not only supports cancer growth but exerts a clinically relevant selective pressure for cancer progression. The assumption that cancer development is accompanied by a profound alteration in neighbor tissues is a new angle in oncology timeline [5]. The hypothesis of an active role of the TME in cancer growth is supported in PCa by at least two types of evidence: (1) the contribution of reactive stroma in PCa carcinogenesis and progression recapitulates the physiologic support of stroma to epithelial cell growth during normal development (embryonic reactivation hypothesis), and (2) the stromal response during PCa growth is similar to the physiological mechanisms associated with regenerative response to an emerging noxa (non-healing wound hypothesis).

According to the first pieces of evidence, it is well known that prostate formation requires paracrine interactions between mesenchymal and epithelial components. Mesenchyme–epithelial interactions play a central role in prostate organogenesis, and some studies demonstrated key similarities in molecular mechanisms involved in prostate development and cancer growth [6,7]. Indeed, tissue recombinant assays clearly demonstrated that urogenital sinus mesenchyme is necessary and sufficient to form prostate tissue in the presence of several different endodermal and ectodermal tissues [8]. Key regulators of branching morphogenesis include FGF, hedgehog, Bmp, TGF-β, Wnt pathways as well as matrix remodeling enzymes, and, importantly, the same factors are recognized to play important roles in carcinogenesis [9].

Secondly, tumor growth activates those pathways that are normally associated with inflammation and tissue healing, involving modification in extracellular matrix, production of de novo growth factors and altered phenotype in stromal cell. Evidence of tumor-induced blood vessel permeability was the basis for the well-known theory expressed in 1986 by H.D. Dvorak that compares tumors to wounds that do not heal [10]. Several chronic features associated with tumor growth can mimic characteristics that are typical of non-healing wounds, including necrosis, apoptosis, hypoxia, extracellular matrix damage and release of cytokines/growth factors. These aspects contribute to activate the wound-healing program triggering functional and phenotypic changes in stromal cells adjacent to tumor mass. A frequently reported response program associated with both wound healing and reactive stroma includes the activation of autophagy. Multiple metabolic stressors, such as hypoxia, nutrient deprivation and degradation of the extracellular matrix (ECM), can activate autophagy both in stromal and tumor cells. Autophagy is considered an inherently cytoprotective mechanism, supplying energy and compounds for cell survival and metabolism. However, autophagy has a paradoxical role during different stages of tumor development and in different cell types, rendering poorly feasible a future application of a specific autophagic signature in clinic prognostic procedures [11].

The challenging questions in this landscape are whether the success of tumor growth is strictly dependent on specific modifications in the surrounding tissue and whether this transformation has a diagnostic or prognostic value. A first answer was proposed by J. Levin following his study about the transplantability of cancers in mice [12]. Interestingly, Levin used the term “reactive stroma” in order to describe modifications in the mouse tissue that received the tumor xenograft, and, currently, the same term is adopted to define the altered characteristics of the TME closely adjacent to tumor mass. Characteristics of reactive stroma include inflammation, hyperplasia, angiogenesis and fibrosis. In addition, reactive stroma has been proposed as an early promotion event in prostate tumorigenesis, and, indeed, mouse model studies in PCa were among the first to show the tumor-promoting role of stroma from tumor-bearing prostate. In fact, it has been demonstrated that the inoculation of non-tumorigenic or low-tumorigenic prostate epithelial cells with mesenchymal cells significantly improved the tumorigenicity in vivo [13]. Ayala et al. developed a semi-quantitative grading scale of the reactive stroma in PCa, in which reactive stroma grade (RSG) 0 was associated with the reactive stroma comprising between 0 and 5% of the tumor area, RSG 1 with 5–15%, RSG 2 with 15–50% and RSG 3 with >50%. The authors showed that a higher level of reactive stromal response is connected to biochemical recurrence (BCR) and increased risk of death due to PCa [14,15]. Several studies have linked high reactive stroma content to a worse clinical outcome, including BCR, development of castration-resistant prostate cancer (CRPC) and prostate cancer-specific mortality [16,17,18,19,20,21].

Although adaptation processes seen in prostate reactive stroma could be involved in many diseases, and not specific for tumors, they offer the plausible biological bases for investigating gene expression signatures also in normal cells present in the TME. Of note, in the presence of tumor cells, prostate stromal cells show a significantly different gene signature with respect to normal tissue [22]. More than three thousand gene expression changes were observed between normal volunteer-derived prostate biopsy samples and stroma from near tumors. Among the most common and relevant differentially expressed genes, numerous were associated with expression in nerve and muscle. In addition, the evaluation of biochemical associations revealed upregulation in specific pathways, including TGF-β axis and Wnt pathways. The diagnostic application of the stroma-specific signature was also able to predict the tumor status of patients [23]. Another expression signature study found significant upregulation in specific processes, such as neurogenesis and axonogenesis, beside alterations that could be associated with wound-healing pathways, including DNA damage/repair and stemness. Different gene expression signatures were identified in stroma adjacent to high-grade and low-grade cancer, confirming the role of the microenvironment in guiding tumor progression. In addition, the prostate stroma gene expression profiling demonstrated a tumor-specific adaptation able to drive tissue-specific metastatic progression. In this context, bone remodeling pathways were upregulated in xenograft models and in high compared to low-Gleason grade cases, suggesting the importance of selective pressure by PCa stroma in conditioning tumor cell osteotropism [22,24]. Accordingly, profiling of stromal gene expression led to development of a stroma-derived metastasis signature that was validated as independently prognostic for the metastatic potential of PCa. A total of 124 differentially expressed stromal genes were identified in PCa patient-derived xenograft models and include genes involved in the modulation of cell–cell and cell–matrix interactions (such as galectin1 and 3, osteopontin and Adam12) [25]. Collectively, data accumulated from gene expression analyses suggest a profound modification of tumor stroma with respect to normal stroma, which supports the importance of cancer–stromal interactions during the process of PCa growth and metastasis. In the TME, mesenchymal cells play pivotal roles in PCa progression and metastasis [26,27,28]. Recently several studies demonstrated a complex spatial and functional heterogeneity of the TME-associated mesenchymal cells during PCa progression by using scRNA sequencing [29,30,31]. Stromal cell-mediated autocrine and paracrine mechanisms influence the structure and function of the TME during disease progression [32,33]. Congruently, it is recognized that stromal AR (androgen receptor) signaling affects the phenotype of prostate epithelial cells at different stages of PCa progression [26,34,35]. During PCa progression, stromal AR signaling avoids progression toward a more aggressive status (e.g., neuroendocrine status). Furthermore, Pakula et al. demonstrated that low AR expression is correlated with high expression of periostin, reduced overall survival, higher Gleason score and neuroendocrine status in PRN mouse model [36,37,38,39].

**Figure 1:**
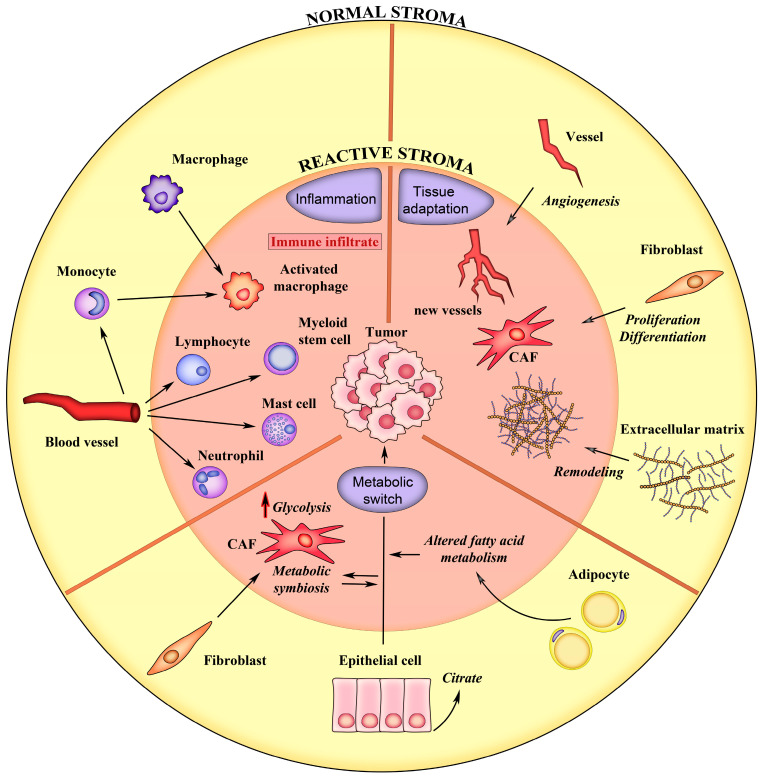
Schematic representation of the main events associated with the formation of reactive stroma in prostate cancer. The normal tissue characteristics are depicted on yellow background. The processes of the reactive stroma are depicted on pink background. See text for details.

In the following chapters, we describe PCa stroma modifications utilizing three main categories: 1—tissue adaptation, including alterations in the ECM and in cells that regulate its homeostasis; 2—inflammatory response and immune cells activation; and 3—metabolic reprogramming, including alteration in energy metabolism ensuing from the synergy between normal and cancer cells (Figure 1). These processes deeply contribute to PCa initiation, progression and response to treatment, and the identification of process-specific biomarkers could potentially contribute to discover a new generation of precision biomarkers.

## 2. Tissue Adaptations

With the denomination of “tissue adaptation”, we collectively refer to phenotypic changes in stromal resident cells in consequence of adjacent tumor growth. The non-epithelial tissue of the prostate comprises primarily smooth muscle cells and less-abundant populations of fibroblasts, vascular cells, nerve cells and non-resident infiltrating immune cells. As previously described, during carcinogenesis and tumor progression, prostate stroma could undergo tissue adaptations typical of chronic wounds. These adaptations include formation of new blood vessels, differentiation of fibroblasts and ECM remodeling [40,41].

### 2.1. Angiogenesis

Blood capillary architecture, and in particular microvessel density (MVD), could offer important hints in the diagnostic procedure. MVD count does not vary significantly in physiological condition; on the contrary, angiogenesis is an early requirement for the maintenance of tumor growth. The increase in MVD offers important physiological advantages to tumor growth, including the necessary oxygen and nutrient supplies. However, endothelial cells could play a direct role in PCa progression, and, in particular, the tissue expression of biglycan, a factor specifically released by vessels, has demonstrated a significant relationship with progression to CRPC [42]. The level of new vessels in tumor and peri-tumor tissue was frequently analyzed by the evaluation of vessel density after staining of specific markers for endothelial cells. The immunohistochemical staining for vWF, CD31, CD34 and CD105 to measure MVD was suggested to be a reproducible method for characterizing the individual tumor [43,44,45,46]. Indeed, MVD is significantly higher in cancer tissue than in the adjacent benign and hyperplastic tissue and it increases toward the center of the tumor [47]. Over the past ten years, research has posited that MVD serves as a useful prognostic indicator in the context of PCa. In addition, it also provides insights into the extent of angiogenic activity within PCa tumors. Employing MVD scoring is recognized as a valuable, straightforward and practical histological technique for the routine evaluation of PCa. Furthermore, MVD has been identified as a reliable forecaster of BCR in PCa patients. In particular, cases of PCa in the earlier T stages with high MVD show a significant correlation with BCR [48].

High-grade prostatic intraepithelial neoplasia (HGPIN) and atypical small acinar proliferation (ASAP) are the most likely precursors of PCa, as demonstrated by the high percentage of new adenocarcinoma diagnoses following repeat biopsies in presence of these conditions [49]. In addition, Proliferative Inflammatory Atrophy (PIA) has been hypothesized to be a precursor lesion of PIN because it is preferentially observed in the peripheral zone of the prostate where PCa arises more frequently, and, furthermore, it shows a similar chromosomic instability to PIN and PCa [50]. PIN is a precancerous lesion in which some luminal cells of the prostate epithelium start to look and behave abnormally. They exhibit enlarged nuclei and nucleoli and increased abnormal proliferation [49,51]. Although PIN itself is usually asymptomatic, it is considered a precursor of PCa. It is often discovered in biopsies taken when PCa is suspected, and it harbors many of the genetic alterations classically present in PCa [52]. Once HGPIN is detected in a prostatic biopsy, the risk of finding PCa in a subsequent biopsy is 15 times greater than in biopsies without PIN [53,54]. The PIN lesion areas have been frequently reported to express an MVD similar to BPH and lower than carcinoma [55,56]. However, also in PIN, the stroma immediately adjacent to the epithelial basement membranes may contain a richer network of capillaries, in agreement with the evidence of strong immunoexpression of VEGF in HGPIN [55]. In addition, as demonstrated by Montironi et al., a deeper analysis of blood capillary permits to individuate early modification in MVD with capillaries that appeared located in the proximity of the basement membrane of ducts and acini [57]. During the progression from HGPIN to adenocarcinoma, an increase in shorter capillaries with open lumen and greater number of endothelial cells were also observed. This suggests that, beside MVD, the morphology of capillaries could represent an early marker of cancer progression. However, quantitative immunohistochemistry of PIN lesions demonstrated that they acquire a significant increase in MVD with respect to benign lesions only in the presence of a relatively widespread fragmentation of the basement membrane [58]. The constant transformation of vascular morphology toward aberrant network is certainly a confounding variable and a likely underlying reason for a lack of a statistically significant association between MVD and PCa stage, condition reported in some studies. This difficulty is augmented by the difference in pathological significance and prognostic roles of different markers of endothelial cells (Table 1) [59].

The activation of angiogenesis in tumor tissues can also be evaluated by analyzing the expression of vascular growth factors and their receptors. VEGFs are angiogenic growth factors that play an important role in PCa progression [60]. They are secreted by cancer cells, macrophages, fibroblasts and mast cells [61,62,63]. VEGFs produced by myofibroblasts promote the accumulation of macrophages at site of fibrosis, that in turn induces angiogenesis and invasion. The expression of VEGFs in PCa is associated with advanced clinical stage, Gleason score, pathologic tumor stage, progression, metastasis and poor survival [64,65,66]. The inhibition of VEGF-A blocked angiogenesis and tumor growth, supporting the role of VEGF-A in PCa progression and its therapeutic use for treating patients with advanced PCa [67,68,69]. Among all the VEGFs, the most predominant growth factor, overexpressed in PCa, is VEGF-A [64,70]. High expressions of VEGF-A and VEGFR-2 in the stroma were independently associated with higher incidence of biochemical failure [46]. Additionally, these two factors have been identified as markers indicative of PCa cases with elevated risk of cancer progression [71].

On the contrary, VEGF-D and VEGFR-3 expressions were significantly reduced in tumor stroma compared to benign tissue, suggesting a prognostic role for lymphangiogenesis [72]. However, differential expression of VEGF ligands and receptors has been associated only sporadically with metastatic spread.

Overall, it is widely accepted that histological biomarkers, able to predict angiogenesis, could be useful prognostic indicators [73,74].

### 2.2. Cancer-Associated Fibroblasts

In the prostatic stroma, fibroblasts are responsible for maintaining the integrity of the epithelial cells by secreting precursors (i.e., collagen type I and III, fibronectin) of the ECM and paracrine factors [75]. Several studies demonstrated that cancer-associated fibroblasts (CAFs) play an important role during prostatic neoplastic transformation creating the reactive stroma through the crosstalk with PCa cells [76,77,78]. CAFs are a heterogeneous cell population that controls the homeostasis of prostate ECM, and their contribution to PCa initiation and progression was demonstrated in several studies. Establishment of a reactive stroma is already observable in PIN, including through differentiation of normal fibroblasts and myofibroblasts surrounding the lesions [79]. The functions of CAFs also evolve along with cancer progression, and, for example, as demonstrated in an orthotopic spheroid culture xenograft model, patient-derived CAFs stimulated metastatic spreading [80]. Prostate fibroblasts’ transition toward CAFs generates a stable phenotype that is considered the result of epigenetic alterations, particularly enriched at regulatory regions of the genome [81]. A large set of differentially methylated regions are shared across CAFs from PCa at different stages, and a specific methylation signature can be utilized to discriminate aggressive PCa from moderate-risk patients. Interestingly, EDARADD gene that showed the greatest difference in methylation in high-Gleason grade cohort was associated with defective paracrine interactions between stroma and epithelium during aberrant development of ectodermal tissues [82].

The heterogeneity renders difficult to precisely characterize CAF phenotype; thus, they are defined by a combination of morphology, tissue position, lack of lineage markers that are expressed in other cell types and fibroblast subtype markers. However, the majority of the studies revealed that the prevalent phenotypic transition in cancer-associated stroma is the presence of myofibroblasts, characterized by expression of smooth muscle α-actin (α-SMA). CAFs make up approximately 50% of reactive stroma in Gleason 3 focus and are progressively replaced by myofibroblasts in Gleason 4 to 5 foci [79]. Also, vimentin and platelet-derived growth factor receptor-α (PDGFR-α) expressions were suggested as predictive markers of myofibroblastic transition associated with PCa progression [83]. At the same time, a decrease in calponin in the TME could be indicative of a reduction in the presence of smooth muscle cells [79].

Significant examples of early reactive stroma derived from transgenic models for spontaneous PCa are present in the literature [84]. In fact, also considering the histologic differences resulting in a thinner fibromuscular stroma in mouse prostate with respect to human prostate in the TRAMP model, it is evident that a stromal response adjacent to moderately well-differentiated acini is detectable [84]. In mouse stroma, beside smooth muscle cells (CD34^+^/SMA^+^), another three types of fibroblasts were found: subepithelial cells, wrapping cells associated with smooth muscle cells and interstitial fibroblasts [85]. In probasin-large T antigen transgenic (LPB-Tag) mice, a model recapitulating tumorigenesis, reactive stromal proliferation was induced and continued to develop throughout the progression to high-grade dysplasia, carcinoma in situ and adenocarcinoma [86]. Immunohistochemical analyses in LPB-Tag indicated that most stromal cells stained positively for α-SMA, suggesting that hyperplastic stroma is determined by mesenchymal cells that had differentiated into smooth muscle cells. However, beside procedures based upon few reliable antibodies, to date, the accurate recording of CAF numbers and subtypes within clinical samples is challenging, and future diagnostic procedures involving CAF phenotyping will potentially benefit from standardization of multiplex technology [87].

The presence of TGF-β in vitro was sufficient to induce a stromal reaction characterized by differentiation of fibroblasts to myofibroblasts [88]. TGF-β mediates the transition of fibroblasts to CAFs and promotes angiogenesis and metastasis [89]. Phenotypic transformation associated with a new secretome was demonstrated in mouse prostate reconstitution (MPR) models where desmoplastic promotion activity was associated with the capacity of C57BL/6 mesenchyme to produce elevated TGF-β1 levels [90]. In addition, expression of TGF-β by PCa cells was fundamental in modulating the stromal microenvironment and accelerating the progression of the tumor [91]. TGF-β acts as promoter in early stages of a spontaneous prostate Pten KO tumorigenesis mouse model [92]. Indeed, genesis of the reactive stroma phenotype occurs also in precancerous PIN lesions and may be associated with elevated TGF-β expression by PIN epithelial cells [79]. Interestingly, a loss of TGF-β receptor type II was observed in epithelial but not in stromal cells during PCa development, and this could explain the direct antagonistic role of TGF-β against cancer cells during progression [93,94]. Although many studies reported decreased levels of TGF-β in PCa [95], others explained that PCa patients with higher Gleason scores showed elevated expressions of TGF-β, indicating that this factor could be a prognostic biomarker of PCa progression. In particular, CXCL13 production induced in myofibroblasts by TGF-β, in castration-resistant mouse models, was fundamental in the emergence of more aggressive cancer types [96]. Due to the complexity of TGF-β signaling in prostate TME, further studies are needed to better understand the role of TGF-β in prostate carcinogenesis and its potential use as a diagnostic target.

Beside upregulation of TGF-β expression, tumor-associated reactive stroma involve other signaling pathways normally associated with wound repair, including FGF and Wnt. PCa stroma secrete Wnt proteins that activate Wnt signaling in tumor cells and promote therapy resistance and disease progression. Wnt/β-catenin signaling is an essential mechanism underlying myofibroblast differentiation [97]. Wnt3a promotes the formation of a myofibroblast-like phenotype in cultured fibroblasts, in part, by upregulating TGF-β signaling, whereas studies in mice show that stromal Wnt3a activates canonical Wnt signaling in the epithelium, facilitating progression of PIN lesions to adenocarcinoma [98,99]. Moreover, Wnt is involved in neuroendocrine differentiation and CRPC cells as a consequence of the release of SFRP1 by endoglin-positive CAFs [96,100]. Increased mRNA expression of several secreted members of Wnt family, including Wnt2, Wnt4 and Wnt9a, have been associated with the development of multi-focal hormone-sensitive PIN lesions in an epigenetic modulated mouse model. In the same mouse model, overexpression of Wnt antagonists in the stroma strongly suppressed the development of precancerous lesions [101]. The knockdown of Wnt10B in prostate stromal cell lines resulted in decreased tumorigenesis in a xenograft model containing LNCaP cells. In addition, in stromal cells, the knockdown determined in stromal cell downmodulation of multiple cytokines such as VEGF-A, Bdnf and CSF2 [102].

Autochthonous transgenic mouse models demonstrated that Wnt signaling alone in the stroma is associated with a reactive stroma phenotype in synergy with FGFR1-induced proliferation and pre-malignant transformation [103]. FGF family members and FGF receptor variants represent key factors in the two-way stromal–epithelial cell dialogue in premalignant lesions. The FGF family contains 18 secreted members that are grouped into six subfamilies based on sequence homology. Different stromal subtypes express specific FGF patterns, and this correlates with potential heterogeneity in the stromal response and tumorigenesis. Prostate stroma secretes FGF-2, FGF7 and FGF10, and they exert a potent mitogenic activity on epithelial cells [104]. Stromal FGF-2 expression is detectable in a large proportion of advanced PCa where it is associated with adverse clinicopathological features and chromosomal instability in tumor cells [105]. Previously, it was demonstrated that stromal FGF-2 expression and release were critical in a xenograft model in sustaining TGF-β-induced PCa growth and MVD [106]. Importantly, FGF7 and FGF10 are expressed in stromal cells but absent in epithelial cells. They are involved in prostate organogenesis and bind the receptor FGFR2IIIb exclusively expressed by epithelial cells, where it has a suppressive role on proliferation [107]. Fibroblasts mainly expressed FGF10 and FGFR1, whereas FGFR3 was detected only in α-actin-positive stromal cells [108]. FGF1, that is expressed at only trace levels in normal adult prostates, was also upregulated in the stroma of malignant tumors that evolve from premalignant, but this evidence was observed only in a murine model of PCa [109]. Recently, Ruder and collaborators, using digital image analysis and a machine learning approach, developed a biomarker of the prostate stroma called quantitative reactive stroma (qRS) [110]. qRS is a measure of percentage of tumor area with a distinct reactive stromal architecture. This algorithm was coupled with Kaplan–Meier analysis to determine survival in a large retrospective cohort of radical prostatectomy samples, supporting the idea that qRS could be a potential biomarker able to add information on the standard predictive models.

### 2.3. Extracellular Matrix

ECM is a fundamental determinant of tissue homeostasis both in health and disease. Prostate ECM is composed of a mixture of collagens, glycoproteins laminins, fibronectins, tenascins and hyaluronan. ECM remodeling is vital for wound healing allowing the migration of cells and modulating cell phenotype. These mechanisms are possible through the ability of cells to bind ECM proteins, activating specific signal transduction pathways. Frequently, changes in the composition and architecture of ECM associated with different pathophysiologic events are determinant in guiding the adaptation of the tissue [111]. It is well known that tumors benefit by an inherently altered ECM with respect to normal tissue, and aberrant ECM is considered an important risk factor for tumor initiation and metastasis [112]. Significant ECM alteration was frequently reported in PCa from early stages to metastatic disease [113]. Dynamic changes in the ECM due to CAF activity could be mainly referred to as “desmoplasia”. In fact, ECM remodeling in PCa progression is associated with an increased collagen deposition and stiffer matrices, characteristics that have been associated with more aggressive cancer cell phenotypes and resistance to therapy [114]. Tumor tissue stiffening was proposed as an early event in PCa tumorigenesis, and increased type I collagen synthesis was observed in activated periacinar fibroblasts adjacent to PIN [79]. Interestingly, PCa cells implanted orthotopically demonstrated accelerated tumor growth in aged compared with young mice. Indeed, the immunohistochemistry analysis of tumors from aged mice revealed a substantial increase in collagen fibers compared with tumors in young mice [115]. A possible explanation for an increased fibrotic response to cancer cells in aged mice is a reduced ability by stromal cells to maintain the structure and composition of ECM [116]. Ultrasound shear wave elastography confirmed collagen remodeling around localized PCa and showed characteristic changes according to Gleason grade. In particular, when comparing the collagen orientation, it was evident that collagen fibers became more oriented as the PCa became more aggressive [117]. In murine models, it has been shown that radial alignment of collagen fibers relative to tumor mass facilitates invasion and thus cancer progression [118].

Beside collagen deposition and remodeling, CAFs produce other potentially prognostic extracellular matrix glycoproteins, including fibronectin and tenascin-C (TNC). Fibronectin polymerization is a critical regulator of ECM organization and stability, and this implicates a potential role for fibronectin in mediating PCa progression. However, available data are contradictory; in fact, whereas fibronectin-rich ECM guides cancer cells to migrate directionally, in advanced PCa, the expression pattern of fibronectin was sporadic or reduced [119]. High expression of TNC has been associated more consistently with poor prognosis in a variety of malignant tumors [120]. TNC was among the most abundant stromal genes upregulated in patient-derived PCa xenografts and highly expressed in castration-resistant models [24]. In vivo studies demonstrated that PCa cells induced stromal reprogramming associated with TNC secretion and that TNC knockdown decreased metastasis to bone [121]. The evaluation of TNC expression via immunohistochemistry in a large cohort of PCa tissue samples confirmed its clinicopathological relevance and its correlation with poor prognosis in PCa patients [122].

Asporin (ASPN) is an extracellular secreted protein initially identified in human cartilage. Asporin was also detected in PCa stroma, but not in benign tissue, and is positively associated with reactive stroma and disease progression [123]. Interestingly, polymorphisms in the D-repeat-sequence variants (ASPN-D) were reported to favor PCa progression and metastatic recurrence, data that were confirmed also in orthotopic xenografts [124].

Periostin is an extracellular matrix glycoprotein frequently detected in several tissues during tissue repair and regeneration. Whereas periostin expression was weak or absent in normal prostates, stronger staining was found in peritumoral stroma. A positive correlation between total periostin staining and Gleason score was observed in different studies [125]. In addition, high stromal periostin was significantly associated with shortened PSA relapse free survival times. Interestingly, circulating periostin was also proposed as a useful marker in improving predictive values of clinicopathological variables [126].

Stromal cells significantly contribute to remodeling TME also by releasing extracellular matrix proteases, such as metalloproteases (MMPs). MMPs are a multigene family of zinc-dependent endopeptidases that share similar structures and the ability to virtually degrade all components of the ECM, playing a central role in morphogenesis, wound healing, tissue repair and remodeling to deal with injuries [127]. Some studies have also shown that MMPs are correlated with Gleason score, disease-free survival, tumor recurrence and other factors related to PCa [128,129,130]. MMP2 and MMP9 stand out because their biomarker potential has been frequently studied and documented in the literature [131].

Indeed, MMP2 is expressed by stromal cells in more than 70% of PCa tissues analyzed [132], and Murray et al. suggested that its expression in bone marrow micrometastasis is associated with worse prognosis in PCa patients after radical prostatectomy. While similar findings sustain a potential role of MMP9 as biomarkers in PCa, current data are still ambiguous and do not support the use of metalloproteases as PCa biomarkers [132,133,134,135].

Tissue inhibitors of matrix metalloproteinases (TIMPs) control MMP activity [136]. The unbalance between TIMPs and MMPs was observed during PCa progression and metastasis [136,137]. In addition, the treatment and prognosis of PCa are strictly dependent from the balance between these two molecules. A recent study demonstrated that TIMP-1 can be used as a biomarker to decide the best treatment for patients with metastatic prostate cancer [138]. These findings suggest that MMPs and TIMPs could be used as potential prognostic biomarkers for patients with PCa, but further investigations are needed [136,139].

Recently, Pu and collaborators demonstrated that elevated monoamine oxidase B (MAOB) in stromal fibroblasts contributes to PCa progression by promoting a reactive stroma, through enhanced ROS production and increased levels of stromal markers (i.e., *TGF-B1*, *VIM* and *FAP*), and favoring the interaction between stromal and epithelial cells via CXCL12/CXCR4 signaling activation [140]. In fact, pharmacological inhibition of stromal MAOB reduces tumor growth in vivo. Additionally, high expression of MAOB in tumor stroma is associated with poor clinical outcomes in patients with PCa. Given the important role of stromal-derived MOAB in PCa tumor progression and invasion, it could be used as a new biomarker to improve the prognosis of PCa patients. The inhibition of MAOB could be a potential new strategy to counteract PCa tumor progression and ameliorate the clinical outcome of PCa patients [140].

## 3. Prostate Cancer Metabolic Reprogramming: The Contribution of TME

Prostate gland shows a unique metabolism based on the elevated production and secretion of citrate, a major component of the prostatic fluid and a key factor for sperm viability [141]. To support citrate production, healthy prostate cells have a truncated tricarboxylic acid (TCA) cycle and do not oxidase this metabolite that instead represents the final product of the Krebs cycle. To this end, high levels of zinc are shuttled into the mitochondria from the extracellular space via SLC39 transporter, whose expression is regulated by androgen receptor (AR) signaling. Accumulated zinc inhibits (m)-aconitase (ACO-2), the enzyme responsible for citrate oxidation. Therefore, prostatic epithelial cells are characterized by reduced levels of oxidative phosphorylation (OXPHOS), with a 65% reduction in the production of ATP via TCA compared to citrate-oxidizing cells [141].

During oncogenic transformation, PCa cells undergo profound changes in their metabolism. Primary PCa shows enhanced OXPHOS [142,143,144,145,146]. In addition, a lipogenic phenotype, characterized by increased de novo lipogenesis (DNL), has been observed at early stages of PCa and further enhanced during cancer progression [142,143,144,145,146,147,148,149]. Differently from the majority of solid cancers, primary PCa is less glycolytic, as confirmed by the low uptake of 18-fluorodeoxyglucose (FDG) [150]. On the contrary, glycolytic metabolism becomes fundamental in advanced CRPC, along with amino acid metabolism, mainly in the form of glutamine anaplerosis into the TCA cycle [151,152,153]. Glutamine is taken up by PCa cells and activates mTOR signaling, favoring their neuroendocrine differentiation [154].

AR signaling has been recognized as the major molecular driver of PCa progression and many pieces of evidence showed how this hormone-sensitive transcription factor is crucial to rewire PCa metabolism toward OXPHOS and DNL during oncogenic transformation by regulating several downstream effectors, including mitochondrial pyruvate carrier (MPC), estrogen-related receptor ERRγ, μTOR and fatty acid synthase (FASN) [145,146,148,155,156]. The reactivated AR signaling is also crucial in CRPC setting, where elevated AR-dependent lipogenic gene signatures (*FASN*, *long-chain acyl-CoA Synthetase 3 (ACSL3)*, *Membrane-bound O-acyltransferase domain containing protein 2* (*MBOAT2*) and *fatty acid elongases 5 and 7* (*ELOVL5/7*)) and increased expressions of the glucose transporter 1 (GLUT1) are associated with poor patient outcome [151,152,157,158]. However, the metabolic rewiring of PCa cells is not exclusively dependent on cell-autonomous mechanisms but is also strongly influenced by surrounding TME.

High reactive stroma content in PCa tissues, characterized by high amounts of CAFs and proinflammatory immune cells, was associated with a specific metabolic profile linked to increased inflammation and ECM remodeling, including low levels of citrate and spermine and high levels of leucine (Table 1) [21]. It is well recognized that CAFs establish a metabolic symbiotic cooperation with PCa cells through lactate shuttle that fuels tumor progression [159]. Human prostate fibroblasts undergo Warburg effect upon exposure to PCa-conditioned media, thus becoming PCa-activated fibroblasts, characterized by increased expression of glucose transporter 1 (GLUT1), elevated glucose uptake and extrusion of lactate. In turn, the CAF-secreted lactate is uploaded by PCa cells to fuel both OXPHOS and anabolic pathways supporting cell proliferation and tumor growth in glucose-free milieu. This metabolic coupling between CAFs and PCa cells involves the overexpression of (i) lactate monocarboxylate transporter 4 (MCT4) in CAFs, responsible for lactate extrusion in the TME and (ii) MCT1 in PCa cells, which is the main transporter for lactate uptake [159]. Notably, both MCT4 and MCT1 levels have a prognostic relevance in PCa. Higher MCT4 expression in the stroma of human PCa has been associated with advanced tumor stages and poor clinical outcomes [160,161]. Likewise, MCT1 expression and lactate uptake have been observed in p53-null PCa, with elevated levels of MCT1 being found in patients with worse prognoses [162]. Moreover, when the expression of MCT1 and MCT4 in PCa cells and surrounding stroma was explored in a large cohort of patients, the elevated co-expression of MCT1 in tumor and MCT4 in stroma was found to be independently associated with a worse biochemical failure-free survival, and the strength of this association was as strong as having a Gleason score [163].

Another key modulator of the lactate-dependent metabolism displayed by PCa cells upon CAF exposure in highly infiltrated cancers is the pyruvate kinase PKM2 isoform, the rate-limiting enzyme of the last step of glycolysis. In vitro studies showed that the metabolic activity of PKM2 in PCa cells is inhibited by CAF conditioning through the induction of PKM2 oxidation and phosphorylation. In this way, PCa cells are forced to shut down glycolysis and switch to CAF-derived lactate-driven OXPHOS. In addition, this metabolically inactive form of PMK2 is able to translocate to the nucleus, where, in association with hypoxia-inducible factor (HIF-1) and the transcriptional repressor Differentially Expressed in Condrocytes 1 (DEC1), it drives the epithelial–mesenchymal transition (EMT) program by repressing the expression of miR-205 [164]. Accordingly, targeting PKM2 nuclear translocation by DASA-58 destroyed the CAF–PCa cell metabolic coupling by restoring glucose dependency and impaired metastatic spread. The clinical relevance of this CAF-dependent PMK2/OXPHOS/EMT axis was confirmed by finding nuclear localization of PMK2 in tumor specimens with higher Gleason scores (8–10) and cancer aggressiveness.

Recently, Ippolito et al. identified the axis sirtuin 1 (SIRT1)/peroxisome proliferator-activated receptor gamma coactivator-1 (PGC-1α) as a crucial player in the CAF-dependent mitochondrial re-education of PCa cells and a marker of PCa invasiveness [165]. Upon CAF-derived lactate uptake, PCa cells experience an unbalanced NAD^+^/NADH ratio, which in turn caused the SIRT1-dependent activation of PGC-1α, a key regulator of mitochondrial biogenesis and OXPHOS. Accordingly, mitochondrial activity and mitochondrial mass were both increased in CAF-exposed PCa cells. Moreover, CAF-reprogrammed PCa cells showed an increased oxygen consumption rate (OCR). This elevated exploitation of mitochondria also resulted in (i) TCA deregulation, (ii) accumulation of oncometabolites like succinate and fumarat and (iii) increased production of mitochondrial reactive oxygen species, all events that drove CAF-induced EMT signature in PCa cells. Beside this lactate-dependent mitochondria reshape, highly glycolytic CAFs were also proven to donate their dispensable mitochondria to PCa cells through the formation of cellular bridges, thus further enhancing OXPHOS addiction of cancer cells and ultimately their malignancy [165].

A glutamine-mediated paracrine reprogramming of PCa cells has been also reported by Mishra et al. [154]. By performing a DNA methylome analysis, the authors found the epigenetic repression of the Ras inhibitor *RASAL3* in prostatic CAFs and showed how this epigenetic alteration initiates a cascade of stromal–epithelial interactions. Indeed, *RASAL3* epigenetic silencing promoted the Ras-dependent macropinocytosis for the uptake of albumin, which was in turn degraded to generate glutamine. Stromal glutamine was then shuttled into PCa cells via the increased expression of the glutamine transporter SLC1A5 where it was converted to glutamate by glutaminase (GLS) to replenish the TCA cycle and support the energetic need of proliferating cancer cells. Moreover, CAF-derived glutamine was necessary and sufficient to promote PCa neuroendocrine differentiation via activation of the nutrient sensor mTOR. Accordingly, genetic or pharmacologic blockade of either SLC1A5 or GLS impaired ATP production, cell proliferation and neuroendocrine differentiation observed in CAF-activated PCa cells. The authors found that epigenetic modifications in prostatic CAFs induced stromal–epithelial interactions that, in turn, promoted PCa progression and resistance to androgen-deprivation therapy (ADT). Notably, ADT further promoted *RASAL3* epigenetic silencing and enhanced glutamine secretion by CAFs. Accordingly, elevated glutamine blood levels were found in the cohort of patients showing resistance to ADT compared to the ADT responder group, suggesting that glutamine plasma levels can be used as prognostic biomarkers for monitoring ADT response and development of resistance [154].

Reduced expression of sequestrosome 1/p62 in the stromal component of PCa is also associated with progression to the most aggressive stages [166]. Indeed, while p62 normally acts as a suppressor of inflammation, its loss promotes the instauration of the CAF proinflammatory phenotype, associated with increased expressions of IL-6 and TGF-β, which in turn drives tumor progression. Valencia et al. further showed that loss of p62 was associated with lower levels of reduced glutathione (GSH) and the consequent accumulation of ROS, which in turn were required for IL-6 overexpression. Accordingly, p62-depleted CAFs showed decreased NADPH/NADP ratio, as consequence of a lower glycolytic rate that fails in maintaining NADPH via pentose phosphate pathway. p62 loss was also associated with reduced glutamine consumption and the downregulation of glutamine and cysteine transporters, including SLC7A11, responsible for cysteine uptake, a rate-limiting step in glutathione synthesis. This metabolic/inflammatory reprogramming in p62-lacking stromal cells was due to the inhibition mTORC1/c-Myc axis, which is instead activated by p62 in normal fibroblasts to suppress the establishment of the proinflammatory phenotype [166].

Emerging evidence suggested that white adipose tissue-derived cells contributed to CAF population in PCa, as obesity in PCa patients is associated with cancer progression and increased content of CAFs, and the intake of saturated fatty acids has been recently shown to promote PCa progression [167]. Zhang et al. reported that palmitate induces PCa lineage plasticity via paracrine secretion of the Wnt ligand 5 (Wnt5) from CAFs [168]. In this study, the authors showed how in CAFs the palmitate-induced secretion of Wnt5, previously associated with PCa aggressiveness, induction of PCa bone metastasis and poor prognosis [169], was able to activate Sonic hedgehog (HH) signaling in PCa cells. In turn, palmitate/Wnt5 paracrinal-induced HH pathway promoted in prostate epithelial cells the expression of SOX2, well known to be a regulator of cell trans-differentiation to AR-independent basal-like phenotype, which portends to poor prognosis in PCa patients [100,170,171]. Accordingly, inhibition of fatty acid intake by using anti-CD36 antibodies resulted in the reduction in Wnt5 and SOX2 expressions by CAFs and PCa cells, respectively, decreased proliferation and elevated apoptosis in mice fed with high-fat diet and treated with enzalutamide, suggesting a role for saturated fatty acid signaling in mediating cancer progression and therapy resistance [172].

Based on these findings, we can envisage that the deeper understanding of the metabolic interactions between PCa and TME that fuel PCa progression may be crucial in the future to realize the full diagnostic, prognostic and therapeutic potentials embodied in this crosstalk.

## 4. Inflammation

The link between inflammation and cancer is well characterized, and it is widely accepted that most malignant tumors are associated with chronic inflammation [173]. Inflammation is considered an early event associated with carcinogenesis. PIA is closely associated with chronic inflammation and describes a frequently observed lesion in prostate biopsies characterized by atrophic glandular structures and chronic inflammatory cell infiltrate, including mast cells and macrophages. The affected epithelial luminal cells exhibit enlarged nuclei, increased proliferation and a reduced apoptotic rate [174].

Further, it has been suggested that tumors can be divided into different prognostic subtypes based on their immune phenotype, i.e., ‘immune inflamed’, ‘immune excluded’ and ‘immune desert’ [175]. Importantly, these immune phenotypes are characterized by different immune cell infiltration patterns in stroma (peritumoral) and epithelium (intratumoral), indicating that the spatial location of infiltrating immune cells is important for tumor biology and patient outcome. While earlier studies of PCa highlighted infiltrating mast cells, T lymphocytes, natural killer cells and macrophages as possible predictors of outcome, conflicting reports exist [176,177,178,179,180,181].

TME frequently comprises immune cells such as macrophages, natural killer (NK) cells, mast cells and lymphocytes that secrete multiple biomarkers (i.e., cytokines, chemokines and growth factors) able to fuel carcinogenesis [182,183]. In the TME, macrophages represent the principal type of immune cells and are involved in tumor promotion and progression [184]. In addition, the expression of tumor-associated macrophages (TAMs) is also associated with resistance to therapy [185,186]. Macrophages are highly plastic cells that can elicit pro- or anti-tumorigenic effects on cancer cells depending on contextual cues from their environment.

When recruited into the primary cancer site, macrophages can be polarized into M1 or M2 TAMs in response to cytokine concentrations in the TME. Classically activated macrophages (M1) exert anti-tumoral activities by secreting IL-1β, IL-12 and TNF-α, while alternatively activated macrophages (M2) fuel tumor growth through immune suppression and promotion of angiogenesis and metastasis [187]. Given the presence of M2 TAMs in the prostate tumor milieu, and their correlation with poor clinical outcomes, they could be used as potential prognostic predictors of disease. It is well known that higher presence of M2-like macrophages is correlated with poor clinical outcomes in several malignant diseases including PCa [187,188]. Recently, several genes (i.e., *ACSL1, DLGAP5, KIF23* and *NCAPG*) highly expressed in tumor tissue and correlated with M2 TAMs have been identified as prognostic biomarkers in PCa due to their correlation with poor overall survival [189]. However, additional studies are needed in order to elucidate the role of these genes as potential biomarkers in PCa.

CAFs cooperate to regulate immune response not only with tumor cells but also with immune cells within the TME by secreting several cytokines, chemokines and growth factors [190]. CAF proliferation has been shown to lead to the development of a fibrous stroma, which induces localized vasculature remodeling and a state of hypoxia and chronic inflammation [96].

Several studies demonstrated that crosstalk between CAFs and TAMs during carcinogenesis and tumor progression leads to the generation of a pro-tumoral microenvironment [191]. CAFs produce several factors (i.e., IL-6, CXCL8, TGF monocyte chemotactic protein-1 (MCP-1) and stromal-derived growth factor-1 (SDF-1)) that regulate the macrophage polarization toward the M2-like phenotype and, consequently, their functions [192,193,194,195]. In turn, M2 TAMs influence phenotypic transition of fibroblasts, leading to their transformation into CAFs [196]. It has been shown that CAFs can orchestrate tumor-promoting inflammation via NF-κB activation [197]. In particular, CAFs recruit monocytes via SDF-1, monocyte chemotactic protein-1 (MCP-1), prostaglandin E2 (PGE2), CXC motif chemokine ligand 14 (CXCL14) and CXC motif chemokine receptor 4 (CXCR4) secretion and promote their differentiation toward a M2 pro-tumoral phenotype both in vitro and in vivo [195,198]. Recently, different subtypes of CAFs have been identified based on differential chemokine expressions (i.e., CCL2 and CXCL12) and with their capability to recruit immune cells within the TME [199]. In addition to acting directly on macrophages, CAFs can affect M2 polarization also indirectly by inducing mast cell to secrete interleukin-13 (IL-13) [200].

Yoshihara and collaborators described the crosstalk between stromal and immune cells by using “Estimation of STromal and Immune cells in Malignant Tumor tissues using Expression data” (ESTIMATE) algorithm, which utilizes gene expression data for inferring the proportions of stromal and immune cells in a tumor sample [201]. Furthermore, the functional enrichment of single-sample genes can be predicted using GO and KEGG pathway analyses [202,203]. The authors predicted the levels of infiltrating stromal and immune cells by calculating immune and stromal scores, which formed the basis for calculating the ESTIMATE score of tumor purity for tumor tissues. Zhao and colleagues identified a set of TME-related genes which are predictive of poor prognosis in these patients by using TCGA-PRAD data. The identified list of genes provide a better understanding of this disease and clarify the relationship between prognosis and TME in patients with PRAD [204].

As for TAMs, myeloid-derived suppressor cells (MDSCs), a heterogeneous population of immature myeloid cells, contribute to create an immunosuppressive TME [205]. It has been demonstrated that a subpopulation of CAFs expressing high levels of fibroblast activation protein (FAP) recruits MDSCs via STAT3-C-C motif chemokine ligand 2 (CCL2) signaling, thus supporting a pro-tumorigenic milieu [206]. Furthermore, Wen and collaborators suggested that MDSCs are more susceptible to infiltrate the PCa stromal area rather than the epithelial compartment supporting neoangiogenesis [207]. Additionally, CAFs are able to recruit neutrophils in the TME via IL-6/JAK/STAT3 in various cancer types [208,209].

During tumor progression, the proliferation and invasion of tumor cells as well as the EMT process are controlled by TME-mediated secretion of several growth factors and cytokines including TGF-β, IL-6, IL-8, IL-10 and VEGF [78,210,211,212]. The heterogeneity of secreted molecules is due to the presence of different cell subpopulations within the TME. The transcription factor NF-κB is the principal factor that controls several processes during tumorigenesis by regulating the expression of numerous genes involved in survival, proliferation, angiogenesis and inflammation [213]. It has been demonstrated that constitutive activation of NF-κB is a hallmark of several cancers, including PCa [213,214]. NF-κB is overexpressed in the PCa TME and regulates the expression of numerous tumor-promoting genes including *cyclin-D1*, *IL-6*, *TNFα*, *VEGF*, *IL-8* and *IL-1β* [215,216,217]. The activation of NF-κB in PCa sustains cancer cell survival via activation of anti-apoptotic pathways, angiogenesis and metastasis, indicating that this factor is associated with advanced PCa. Accordingly, NF-κB overactivation also promotes EMT of PCa cells [218], suggesting that NF-κB could be a potential prognostic biomarker in human PCa. Indeed, serum markers of inflammation have been proposed as useful tools for estimating PCa risk and evaluating prognosis [219]. Interestingly, accumulated evidence suggests that PCa inflammatory biomarkers could be detected in urine via non-invasive procedures [219,220].

In PCa, the most important mediator of chronic inflammation is IL-6, a multifunctional cytokine that sustains prostate cancer cell proliferation, inhibits cell death and promotes T cell infiltration to the TME, EMT and metastatic processes both in vitro and in vivo [221,222]. In addition, IL-6 influences the sensibility to androgens via different signaling pathways [223]. IL-6 is secreted by different types of cells including inflammatory and immune cells [224]. IL-6 stimulates PCa cells to secrete VEGF via the activation of STAT3 and MAPK signaling pathways under ADT [210]. Recently, it has been demonstrated that IL-6 promotes the progression of PCa to CRPC via AR activation both in vitro and in vivo [225]. Additionally, elevated IL-6 signaling in PCa samples is associated with advanced stages of diseases and with worse prognoses (Table 1) [226]. IL-6 activates in vitro TGF-β-SMAD2 axis and promotes neuroendocrine differentiation (NED) of PCa cells under androgen depletion conditions [220,227]. Given its important role in tumor progression, as well as the established correlation between high levels of serum IL-6 and shorter survival [228], IL-6 could be a prognostic biomarker for PCa patients. In this context, IL-6 and IL-8 mediate PCa NED through MAPK and STAT3 signaling, supporting their role as potential biomarkers for advanced PCa [227,228,229]. Although no biomarkers able to predict risk of NED are available to date, strong efforts have been made by several groups to investigate the role of secreted protein acidic and rich in cysteine (SPARC) as a potential biomarker. Controversial results are reported due to its expression in the tumor cells and the stromal compartment, but Enriquez et al. unveiled that castration triggers a tumor–stroma crosstalk leading to stromal Sparc downregulation that could be a crucial step for Neuroendocrine prostate cancer (NEPC), supporting its role as a new potential biomarker. Furthermore, although SPARC can limit IL-6 production by a mechanism that is still elusive and needs a deeper investigation, an upregulation of the NF-κB pathway, which in turn promotes IL-6 production, has been observed in Sparc^−/−^ fibroblasts [230,231,232,233].

IL-18 is produced by different immune cells such as B and T cells, NK cells, monocytes and macrophages and controls the immune response [234]. Congruently, IL-18 promotes angiogenesis and metastasis, leading to tumor progression and contributing to immune escape [90,235]. Recently, it has been documented that IL-18 is overexpressed in several cancers including PCa and it is associated with advanced tumor stage and metastasis [236]. Accordingly, high expression of IL-18 correlates with poor prognosis in PCa patients [236]. These findings suggest that IL-18 could be used as a novel prognostic biomarker to identify high-risk patients and as a new potential therapeutic target. Since this cytokine is also produced by other cells, a deeper understanding of the role of IL-18 in PCa will help to better evaluate its prognostic role.

Another important proinflammatory cytokine secreted by inflammatory cells and implicated in PCa progression and castration resistance is IL-8 [237]. IL-8 expression is mediated by NF-κB [238]. IL-8 is also regulated by TGF-β and induces angiogenesis and tumor metastasis [239]. In addition, IL-8 promotes the EMT and the recruitment of lymphocytes to the TME [222]. Also, IL-8 induces prostate cancer proliferation in vitro and stimulates TAMs to secrete growth factors that in turn sustain the proliferation of tumor cells. Accordingly, the inhibition of IL-8 signaling reduces cell proliferation and invasiveness both in vitro and in vivo [240,241,242]. It has been demonstrated that IL-8 is overexpressed in serum of metastatic PCa patients and correlates with poor overall survival [237,243]. Recent studies reported a controversial role of IL-8 expression in association with the expression of androgens [244]; therefore, further investigations are necessary in order to clarify the precise relationship between IL-8 and androgen expression and the potential utility of IL-8 as a prognostic and predictive biomarker in aggressive PCa.

The anti-inflammatory, immune-suppressive cytokine IL-10, secreted by both tumor cells and immune cells, promotes cancer progression by inhibiting the anti-tumor response via different signaling pathways [245]. As for several other cytokines, elevated serum levels of IL-10 are associated with poor prognosis and high Gleason scores in patients with PCa as well as with drug resistance [246]. A growing number of studies are working on the identification of stromal-specific genomic signatures able to predict the progression risk. Among these, Rasmussen et al. identified a specific stromal subtype (using clinicopathological characteristics such as Gleason grade, gene set enrichment analysis and stromal and immune cell infiltration patterns) in primary tumor samples of localized prostate cancer (LPC) and validated their characteristics in two independent cohorts. A specific stromal subtype has been demonstrated to be more aggressive in LPC, showing stromal dysfunction at both the cellular and molecular levels with increased M2-polarized macrophages and CD8^+^ T cell infiltration [247].

## 5. Conclusions

Data from early studies investigating the role of TME in PCa carcinogenesis and progression had a pioneering value in the field of ecology of cancer. Indeed, the hypothesis supporting the role of CAFs in tumorigenesis has been formulated mainly using data obtained in PCa models. However, the clinical translation of this aspect in useful diagnostic or prognostic hints is far from being available. A first problem resides in the high number of modifications evaluable in reactive stroma and in our capacity to discriminate stromal alterations able to support tumor growth from those that are neutral for cancer progression. Indeed, although the cellular and molecular processes involved in the formation of reactive stroma are well defined, cancer-promoting pathways are frequently intertwined with the physiologic defense mechanisms. In addition, it is possible, as seen for cancer cell phenotypes, that a unique promoting pattern of reactive stroma does not exist and that different combinations of molecular characteristics in TME could equally result in a positive effect on tumorigenesis. An example is the metabolic symbiosis between stroma and cancer cells observed only in a subpopulation of PCa patients. Another important weakness is of technical relevance. To date, the gold method for investigating stromal biomarkers is immunohistochemistry, which, however, suffers from different limitations. Among others, we can cite the use of non-standardized antibodies and inter-operator variability. Thus, the future of TME biomarkers will certainly benefit from new high-throughput methodologies that, together with an increased capacity to investigate precisely TME excluding the contribution from cancer cells, will also guarantee a higher reproducibility of the results.

In conclusion, current evidence strongly supports a modern prognostic approach that takes into consideration reference markers associated with the stroma modification and, in particular, with changes in stromal cell phenotype, in ecological metabolisms and in patterns of secreted inflammatory cytokines.

**Table 1:** Potential prognostic biomarkers detectable in reactive stroma. Main biological effects associated with their expression and experimental models used for their validation are also shown.

TME Biomarker	Biological Effect	Experimental Model	References *
*Tissue Adaptation*			
α-SMA, vimentin, PDGFR-α	myofibroblast differentiation	in vivo, ex vivo	[79,86]
Asporin	desmoplasia	in vivo, ex vivo	[123]
Collagen	desmoplasia	imaging in patients	[113,117]
Fibroblast methylation signature	CAF differentiation	ex vivo	[82]
FGF-2	PCa growth, increased microvessel density	in vitro, in vivo, ex vivo	[105,106]
Periostin	desmoplasia	ex vivo	[125]
Tenascin-C	desmoplasia	in vivo, ex vivo	[94,95]
TGF-β	CAF differentiation	ex vivo	[79]
VEGF-A, VEGFR-2	angiogenesis	ex vivo	[70]
vWF, CD31, CD34, CD105	increased microvessel density, aberrant vessel morphology	in vivo, ex vivo	[56,57]
Wnt	CAF differentiation	in vitro, in vivo	[98,99,101]
MAOB	CAF differentiation, ECM remodeling	in vitro, in vivo	[140]
*Metabolic Switch*			
Lactate	metabolic reprogramming of PCa cells toward OXPHOS and anabolic pathways	in vitro, in vivo, ex vivo	[159,160,165]
Leucine	increased inflammation and ECM remodeling	ex vivo	[21,163]
GLUT1	Warburg effect	in vitro	[159]
Glutamine	metabolic reprogramming of PCa cells in the form of anaplerosis into TCA cycle and energy production	in vitro, ex vivo	[154]
MCT4	increased lactate extrusion	in vitro, ex vivo	[160,161]
*Inflammation*			
IL-6	cell proliferation, T cell infiltration into the TME, EMT, metastasis	in vitro, in vivo, ex vivo	[226,228]
IL-8	angiogenesis and metastasis, EMT, T cell recruitment into the TME and castration resistance	in vitro, in vivo, ex vivo	[237]
IL-10	immune escape	in vitro, ex vivo	[246]
IL-18	angiogenesis, metastasis and immune escape	ex vivo	[236]

* Only selected references are indicated. See the text for further pertinent references.
